# A Reaction against Antiseptics

**Published:** 1889-01

**Authors:** 


ARTICLE VI.

### A REACTION AGAINST ANTISEPTICS.

The movement against the use, except in a limited degree, of antiseptics, or, as they are now called, antimycotics, has received strong support from the investigations of Dr. E. Senger (*Deutsche Med. Zeitung*, No. 43, 1888), who shows the dangers of strong antimycotics in surgery, and the inutility of mild ones. Dr. Senger clearly states his position in a discussion following his paper (*London Medical Recorder*): All operations, he said, might be divided into two groups. In the first group strong antimycotics were applied, and these were the less dangerous operations. In the second group weaker solutions, particularly of boric and salicylic acid, were used. Antiseptics were introduced with the intention of killing any fungi that might have settled in the wound; in short, to disinfect. Antimycotic treatment was supported by the classical researches of Koch. Perhaps, however, it would be better, from the practical surgeon's point of view, to establish a basis by means of experiments in the disinfection of pus cocci. No doubt strong antimycotic solutions, such as sublimate and carbolic abid, destroy the staphylococcus aureus, but the weaker solutions do not effect this, though where they are employed there is a greater, nay a fatal, danger from a faulty antisepsis. Just in such cases in which strong solutions should be applied, they are inadmissible on account of the danger of poisoning; and again they are applied when they are much less needed. The theory and practice do not harmonize. To kill a pus coccus that has fallen into the abdominal cavity, washing with a four per cent. solution of boric acid is not sufficient, for you could let the man lie immersed in such a solution for five days and not accomplish that object. The same may be said of salicylic acid. Yet the results of operationa in the abdominal cavity are excellent, and Senger believes that it is only necessary to wash the wound with sterilized water. He lays no particular stress on the common salt solution. The disinfection of a living tissue he considers to be impossible. Either the tissue is free from germs, in which case there is no need of an antimycotic, or it is infiltrated with pathogenic fungi, in which case it is impossible to destroy the fungi without at the same time seriously injuring the tissue. Disinfection answers admirably on dead objects, but in the case of living organs more importance must be attached to vital energy. Did not Virchow, at a time when it seemed as if the whole of pathology depended on pathogenic bacteria, especially direct attention to the conflict between the cells and bacteria? Surgical examination showed this in wounds in connection with syphilis, tabes, diabetes, and other exhausting diseases, so that, in spite of the stronger antiseptics, it was impossible to prevent purulence and purify the wound, because the energy of the cells had much abated and disinfection was impracticable. By all means let dead objects, such as instruments, the operating-room, etc., be thoroughly disinfected, but after the first incision no antimycotic whatever should be used. In how far these theoretical demands can be met in practice must be decided by those who have great opportunities of practice. The latter alone can determine the cases in which antimycotics are indispensable; but the general rule of avoiding their use should be observed.—*Medical Record.*

